# Supplementary material for: Whole-body physics simulation of fruit fly locomotion
Source: Nature. 2025 Apr 23;643(8074):1312–20. doi: 10.1038/s41586-025-09029-4 (PMC12310536; doi:10.1038/s41586-025-09029-4)
Supplement: Supplementary file 1 — This file contains Supplementary Methods, Supplementary Fig. 1, Supplementary Tables 1–21 and Supplementary References. [file 41586_2025_9029_MOESM1_ESM.pdf]

nature portfolio
https://doi.org/10.1038/s41586-025-09029-4

Supplementary information

Whole-body physics simulation of fruit fly locomotion

In the format provided by the authors and unedited

Supplementary Information

Whole-body physics simulation of fruit fly locomotion

Roman Vaxenburg,[1] Igor Siwanowicz,[1] Josh Merel,[2,‡] Alice A. Robie,[1]
Carmen Morrow,[1] Guido Novati,[3] Zinovia Stefanidi,[1,4] Gert-Jan Both,[1]
Gwyneth M. Card,[1,5] Michael B. Reiser,[1] Matthew M. Botvinick,[3,6] Kristin M. Branson,[1]
Yuval Tassa,[3†] Srinivas C. Turaga [1†]

[1]HHMI Janelia Research Campus, Ashburn VA USA

[2]Fauna Robotics, New York City NY USA

[3]Google DeepMind, London UK

[4]Machine Learning in Science, Tübingen University and Tübingen AI Center, Germany

[5]Columbia University, New York City NY USA

[6]Gatsby Computational Neuroscience Unit, University College London, London UK

[‡]Primary contributions while at Google DeepMind

[†]correspondence: turagas@janelia.hhmi.org, tassa@google.com

Contents

1 Supplementary Methods **2**
- 1.1 Constructing the physics fly model in MuJoCo . . . 2
- 1.2 Phenomenological model of fluid forces . . . 3
  - 1.2.1 Kutta lift . . . 4
  - 1.2.2 Magnus force . . . 5
  - 1.2.3 Viscous drag . . . 5
  - 1.2.4 Added mass . . . 6
- 1.3 Flight imitation task configuration . . . 6
- 1.4 Flight controller reuse, vision-guided flight task configuration . . . 8
- 1.5 Walking imitation task configuration . . . 9
- 1.6 Simulation speed . . . 11

2 Supplementary Tables **12**

3 Supplementary References **21**

1 Supplementary Methods

1.1 Constructing the physics fly model in MuJoCo

Here we describe the steps taken in order to create the physics MuJoCo fly model, given the geometrical Blender model and measured masses of fly body parts. The resulting MuJoCo model is available at https://github.com/TuragaLab/flybody.

Initial conversion The Blender-to-MuJoCo export plug-in[1] was used to export a raw MuJoCo model containing only geometrical information: body meshes and a kinematic tree with joint axes and limits.

Model building script The raw MuJoCo model was then loaded and manipulated with a Python script using PyMJCF, a Python library for model manipulation which is part of Google DeepMind's dm_control suite [1]. The following steps were taken.

1. Enforced consistent naming everywhere using a `part_sternum_side` convention e.g., "`coxa_T3_right`". Consistent naming allows for conveniently readable loops like

```
thorax = mjcf_model.find('body', 'thorax')
legs = [body for body in thorax.find_all('body') if 'coxa' in body.name]
links = ['coxa', 'femur', 'tibia', 'tarsus', 'tarsus2', 'tarsus3', 'tarsus4']
sternums = ['T1', 'T2', 'T3']
sides = ['right', 'left']
for sternum in sternums:
  for side in sides:
    for leg in legs:
      if sternum in leg.name and side in leg.name:
        for link in links:
          # Do something with each link in each leg.
```

2. Made use of MuJoCo's cascading defaults mechanism, avoiding repeated values. Common properties like angle ranges, damping and actuator properties for all joints of the same type or masses of links of the same type are given by a single number in the model, defined in a default class, which is then inherited by elements in the kinematic tree.
3. Model units were chosen to be CGS, for better numerical precision. Note that in MKS, some values are extremely small, for example the inertia of a tarsus link is on the order of 1×10^{-20} kg m^2, while in CGS is 1×10^{-13} g cm^2. The difference in accuracy is significant for double-precision floating-point arithmetic.
4. Body moments-of-inertia were computed by MuJoCo given mesh geometries and empirical masses, assuming uniform density for each body part.
5. Kinematic symmetry was enforced to numerical precision, transverse of the sagittal plane.
6. Joint axis orientations were reflected through the saggital plane, ensuring identical semantics on both sides i.e., joint rotation in the positive (negative) direction always corresponds to extension (flexion) and abduction (adduction), respectively.
7. Joint angle reference values were chosen so that 0.0 corresponds to the base pose, see Figure 1 in the main text.

[1] https://github.com/google-deepmind/dm_control

8. A layer of primitive collision geoms was created, initially by letting MuJoCo fit primitives to meshes by matching ineritas, and then by manual fine-tuning.
9. Manually excluded pairs of bodies that cannot collide, both to avoid spurious collisions and to increase simulation speed. Note that certain collisions that are possible in real flies were also excluded. In particular since the modeled wings are rigid and while real wings are flexible, wing-wing and wing-body collisions cannot be well modeled.
10. Added tendons to the abdomen and the tarsi. These "fixed" tendons are a simplification of spatially routed tendons, whose length corresponds to a linear combinations of joint angles, allowing a single actuator to act on multiple joints e.g., to flex the multiple links of the tarsus or abduct the entire abdomen. See examples in Figure 1 in the main text.
11. Set the default body pitch angle to $47.5°$, following [2]. Re-orient the wing joint axes such that when the body is at $47.5°$, the wing yaw axes are strictly vertical and the stroke plane is horizontal.
12. Added a total of 78 actuators:
    - 8 actuators in each leg (coxa: 3, femur: 2, tibia: 1, tarsus: 2), using desired angle (position) semantics, with gains chosen so that a force of approximately one body weight can be applied at the end-effector at the base pose.
    - Wings actuators (yaw, roll, pitch) have torque semantics with gain values of $18.0\ \mathrm{dyn\,cm}$ (also see Methods).
    - 16 additional actuators for proximal joints: head and rostrum (4), haustelli (2), labri (2), antennae (6) and abdomen (2).
    - 6 adhesion actuators at the claws which can apply a force up to $1\times$ body weight.
    - 2 adhesion actuators in the mouth (labrum).
13. Added egocentric sensors (also see Supplementary Table 4):
    - An ideal accelerometer and gyro to the thorax, corresponding to processed vestibular sensor information.
    - An ideal velocimeter, corresponding to processed information from air motion sensors in the hair follicles.
    - Added force and touch sensors at the end effectors. The former report the force passing through the first tarsus joints, while the latter report pressure applied to the claw.
    - Two eye cameras, at the geometric center of the eyes. These are standard openGL cameras with a wide $140°$field-of-view, see main text for how these were used in visually guided experiments.

1.2 Phenomenological model of fluid forces

This section describes the fluid force model we introduced to MuJoCo to facilitate the simulation of fly's flight. The fluid model computes forces exerted on moving rigid bodies whose shape can be approximated by ellipsoids. The model provides fine-grained control of the different types of fluid forces via five dimensionless coefficients, the `fluidcoef` attribute in MuJoCo, see the MuJoCo documentation[2] for more detail. We used the fluid model to compute forces on fly's flapping wings whose shape closely matches slender ellipsoids (Figure 1i in main text). Elements of the fluid force model are a generalization of [3] and [4] to three dimensions. The force $\mathbf{f}$ and torque $\mathbf{g}$ that the surrounding fluid exerts onto the translating and rotating body are approximated as a sum of effects: a lifting force due to the translational circulation f_K, an additional lifting force due to the

[2]MuJoCo documentation: https://mujoco.readthedocs.io/

rotational circulation of the body f_M, a force and torque due to the viscous drag $\mathbf{f}_D$ and $\mathbf{g}_D$, and finally the effect of the added mass $\mathbf{f}_A$ and $\mathbf{g}_A$:

$$\mathbf{f} = \mathbf{f}_K + \mathbf{f}_M + \mathbf{f}_D + \mathbf{f}_A,$$
$$\mathbf{g} = \mathbf{g}_D + \mathbf{g}_A.$$

The MuJoCo model is implemented generally and the forces are referred to, respectively, as Kutta lift, Magnus force, viscous drag and resistance and added mass. We will use this naming convention here. The forces imposed by the fluid onto each ellipsoid are computed independently by approximating the effect of an incompressible quiescent fluid of density ρ and (dynamic) viscosity ν. The problem is described in a reference frame aligned with the principal axes of the ellipsoid and moving with it. The ellipsoid has semi-axes $\mathbf{r} = \{r_x, r_y, r_z\}$, volume $V = (4\pi/3)r_x r_y r_z$, velocity $\mathbf{v} = \{v_x, v_y, v_z\}$, and angular velocity $\boldsymbol{\omega} = \{\omega_x, \omega_y, \omega_z\}$. We will also use $r_{\text{max}} = \max\{r_x, r_y, r_z\}$, $r_{\text{min}} = \min\{r_x, r_y, r_z\}$, and $r_{\text{mid}} = r_x + r_y + r_z - r_{\text{max}} - r_{\text{min}}$. The area projected by the ellipsoid onto the plane normal to $\mathbf{v}$ is

$$A_{\mathbf{v}}^{\text{proj}} = \pi \sqrt{\frac{r_y^4 r_z^4 v_x^2 + r_z^4 r_x^4 v_y^2 + r_x^4 r_y^4 v_z^2}{r_y^2 r_z^2 v_x^2 + r_z^2 r_x^2 v_y^2 + r_x^2 r_y^2 v_z^2}}. \tag{1}$$

The circulation Γ is the line integral of the fluid velocity field $\mathbf{v}_f$ around a closed curve $\Gamma = \oint \mathbf{v}_f \cdot \mathbf{dl}$. The individual force and torque components are described below.

1.2.1 Kutta lift

The Kutta condition describes an effect that is valid also for potential (i.e. inviscid) flows. For a body moving in a potential flow there are two stagnation points (a location in the flow field where the velocity is zero): in the front, where the stream-lines separate to either sides of the body, and in the rear, where they reconnect. The Kutta condition is the observation that a moving body with a sharp rear edge will generate in the surrounding flow a circulation of sufficient strength to hold the rear stagnation point at the trailing edge. In two-dimensional potential flow, the circulation due to the Kutta condition for a slender body can be estimated as $\Gamma_{\text{K}} = C_K \; r_x \; \|\mathbf{v}\| \; \sin 2\alpha$. Here C_K is a Kutta lift coefficient and α is the angle between the velocity vector and its projection onto the surface. The lift force per unit length can be computed with the Kutta–Joukowski theorem as $\mathbf{f}_K/\ell = \rho \Gamma_{\text{K}} \times \mathbf{v}$.

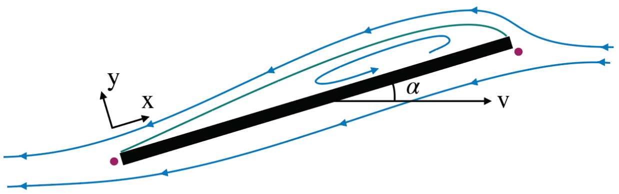

Supplementary Figure 1: Schematic of the Kutta condition. Blue lines are streamlines and the two magenta points are the stagnation points. The dividing streamline, which connects the two stagnation points, is marked in green. The dividing streamline and the body inscribe an area where the flow is said to be "separated" and recirculates within. This circulation produces an upward force acting on the plate.

In order to extend the lift force equation to three-dimensional motions, we consider the normal $\mathbf{n}_{A,\mathbf{v}} = \{\frac{r_y r_z}{r_x} v_x, \frac{r_z r_x}{r_y} v_y, \frac{r_x r_y}{r_z} v_z\}$ to the cross-section of the body which generates the body's projection $A_{\mathbf{v}}^{\text{proj}}$ onto a plane normal to the velocity. We use this direction to decompose $\mathbf{v} = \mathbf{v}_{\|} + \mathbf{v}_{\perp}$ with $\mathbf{v}_{\perp} = (\mathbf{v} \cdot \hat{\mathbf{n}}_{A,\mathbf{v}})\, \hat{\mathbf{n}}_{A,\mathbf{v}}$ ($\hat{\mathbf{n}}_{A,\mathbf{v}}$ is the unit vector). We write the lift force as:

$$\mathbf{f}_{\mathsf{K}} = \frac{C_K\, \rho\, A_{\mathbf{v}}^{\text{proj}}}{\|\mathbf{v}\|} \left(\mathbf{v} \times \mathbf{v}_{\|}\right) \times \mathbf{v} \tag{2}$$

Note that the direction of $\hat{\mathbf{n}}_{A,\mathbf{v}}$ differs from $\mathbf{v}$ only on the planes where the semi-axes of the body are unequal. So for example, for spherical bodies $\hat{\mathbf{n}}_{A,\mathbf{v}} \equiv \hat{\mathbf{v}}$ and by construction $\mathbf{f}_{\mathsf{K}} = 0$.

1.2.2 Magnus force

A spinning body induces rotation in the surrounding fluid which deflects the trajectory of the fluid flow past the body, and the body receives an equal an opposite reaction. Following [3], we estimate the force due to the rotation as

$$\mathbf{f}_{\mathsf{M}} = C_M \rho V \boldsymbol{\omega} \times \mathbf{v} \tag{3}$$

where V is the volume of the body and C_M is a coefficient for the force, which we typically set to 1.

1.2.3 Viscous drag

The drag force acts to oppose the motion of the body relative to the surrounding flow. For high Reynolds numbers, the viscous drag can be approximated with the drag equations, first proposed by Newton, $f_{\mathsf{D}} = -C_D \rho s_D v^2$ and $g_{\mathsf{D}} = -C_D \rho I_D \omega^2$. Here C_D is a drag coefficient, s_D is a reference surface area (e.g., a measure of the area projected on the plane normal to the flow), and I_D is a reference moment of inertia. These quantities depend on the properties of the fluid, the shape of the body and its velocity [5]. We derive a correlation for the viscous drag based on two surfaces: the projection surface $A_{\mathbf{v}}^{\text{proj}}$ of the ellipsoid onto a plane normal to $\mathbf{v}$ and the maximum projected surface $A_{\mathsf{max}} = \pi r_{max} r_{mid}$, such that $A_{\mathbf{v}}^{\text{proj}} \leq A_{\mathsf{max}}$:

$$\mathbf{f}_{\mathsf{D}} = -\rho \left[C_D^{\mathsf{blunt}}\, A_{\mathbf{v}}^{\text{proj}} + C_D^{\mathsf{slender}} \left(A_{\mathsf{max}} - A_{\mathbf{v}}^{\text{proj}} \right) \right] \|\mathbf{v}\|\, \mathbf{v}$$

We propose an analogous model for the angular drag. For each Cartesian axis we consider the moment of inertia of the maximum swept ellipsoid obtained by the rotation of the body around the axis. The resulting components of the moment of inertia are:

$$I_{D,i} = \frac{8\pi}{15}\, r_i\, \max\{r_j,\, r_k\}^4 \tag{4}$$

where, as before, the indices i, j, k are cyclic permutations of the axes (x, y, z), (y, z, x), (z, x, y). The angular drag torque is computed as:

$$\mathbf{g}_{\mathsf{D}} = -\rho \left(\left[C_D^{\mathsf{angular}}\, \mathbf{I}_D + C_D^{\mathsf{slender}} \left(\mathbf{I}_{\mathsf{max}} - \mathbf{I}_D \right) \right] \cdot \boldsymbol{\omega} \right) \boldsymbol{\omega} \tag{5}$$

Here $\mathbf{I}_{\mathsf{max}}$ is a vector with each entry equal to the maximal component of $\mathbf{I}_D$.

For low Reynolds numbers, the viscous drag is well approximated by Stokes' law [6] for an equivalent sphere:

$$\mathbf{f}_\mathsf{V} = -6\pi r_V \nu \mathbf{v} \tag{6}$$

$$\mathbf{g}_\mathsf{V} = -8\pi r_V^3 \nu \boldsymbol{\omega} \tag{7}$$

where $r_V = (r_x + r_y + r_z)/3$ is the radius of the equivalent sphere. We add this term to the previously defined viscous drag force and torques $\mathbf{f}_D$ and $\mathbf{g}_D$ to approximate the drag well for both high and low Reynolds numbers.

1.2.4 Added mass

Added mass measures the inertia of the fluid that is put into motion by the body's motion. In the case of a body with three planes of symmetry, the forces $\mathbf{f}_A$ and torques $\mathbf{g}_A$ can be written as [7]:

$$\mathbf{f}_A = -\mathbf{m}_A \odot \dot{\mathbf{v}} + (\mathbf{m}_A \odot \mathbf{v}) \times \boldsymbol{\omega} \tag{8}$$

$$\mathbf{g}_A = -\mathbf{I}_A \odot \dot{\boldsymbol{\omega}} + (\mathbf{m}_A \odot \mathbf{v}) \times \mathbf{v} + (\mathbf{I}_A \odot \boldsymbol{\omega}) \times \boldsymbol{\omega} \tag{9}$$

Here, $\odot$ denotes an element-wise product, $\dot{\mathbf{v}}$ is the linear acceleration and $\dot{\boldsymbol{\omega}}$ is the angular acceleration. $\mathbf{m}_A \odot \mathbf{v}$ and $\mathbf{I}_A \odot \boldsymbol{\omega}$ are the virtual linear and angular momentum respectively. The added-mass vector $\mathbf{m}_A = \{m_{A,x}, m_{A,y}, m_{A,z}\}$ and added-moment of inertia vector $\mathbf{I}_A = \{I_{A,x}, I_{A,y}, I_{A,z}\}$ measure the inertia of the fluid displaced by the motion of the body in the corresponding direction and can be derived from potential flow theory for certain simple geometries.

For an ellipsoid, the virtual mass $m_{A,i}$ for a motion along axis i and the virtual moment of inertia $I_{A,i}$ for a rotation along axis i are:

$$m_{A,i} = \rho V \frac{\kappa_i}{2 - \kappa_i}, \tag{10}$$

$$I_{A,i} = \frac{\rho V}{5} \frac{(r_j^2 - r_k^2)^2 (\kappa_k - \kappa_j)}{2(r_j^2 - r_k^2) + (r_j^2 + r_k^2)(\kappa_j - \kappa_k)} \tag{11}$$

where the dimensionless virtual inertia coefficients are [8]:

$$\kappa_i = \int_0^\infty \frac{r_i r_j r_k \mathrm{d}\lambda}{\sqrt{(r_i^2 + \lambda)^3 (r_j^2 + \lambda)(r_k^2 + \lambda)}} \tag{12}$$

which we compute by 15-point Gauss–Kronrod quadrature. The indices i, j, k are cyclic permutations of the axes (x, y, z), (y, z, x), (z, x, y).

1.3 Flight imitation task configuration

As the flight reference data, we used previously recorded trajectories of freely flying *Drosophila hydei*. The trajectories contain a fly's Cartesian center-of-mass position and body orientation represented as a quaternion. The trajectories were recorded at 7500 fps. We started with 44 trajectories of spontaneous turns (saccades) [9] and 92 trajectories of evasion maneuvers [2] in response to visual looming stimuli. Each reference trajectory started with the fly first flying normally and then performing a maneuver. During and after the maneuver, the fly could fly straight, sideways, and backwards. The flies could also ascend and descend. We linearly interpolated the raw trajectories to the flight simulation control step of 0.2 ms. Then we augmented (doubled) the dataset by mirroring the trajectories in a vertical plane, taking proper quaternion reflection into

account. This resulted in a dataset of 272 flight trajectories, equivalent to $\sim$53 seconds of real time flight. The dataset is available as Supplementary Data[3]. We used 80% of the trajectories for training and the rest for testing. Due to the small size of the dataset, to maintain balance between left and right turns in the training data, we split the dataset such that if a trajectory is in the training set, so is its mirrored counterpart. We simulated flight at 0.05 ms physics time-steps and 0.2 ms control time-steps (Supplementary Table 15).

The reinforcement learning task is set as follows. In each episode, the fly model is required to track a reference trajectory selected from the flight dataset at random. The episode begins from a random step within the selected reference trajectory, excluding the last 50 steps. The model's initial position, orientation, linear and rotational velocities are set equal to the reference. The initial phase of the wing cycle is randomized. The episode ends either when the end of trajectory is successfully reached, or terminates early if the model hits the ground or is displaced from the reference CoM position by more than 2 cm.

The reward is calculated based on how closely the fly model tracks the reference trajectory at each timestep. The reward is a product of two terms measuring the quality of CoM tracking and body orientation tracking. At every simulation timestep, the reward $R \in [0, 1]$ is computed as:

$$R = \underbrace{\max(0,\ 1 - \tfrac{1}{\delta_{\text{com}}}\|\mathbf{r} - \mathbf{r}^\star\|)}_{\text{CoM displacement}} \times \underbrace{\max(0,\ 1 - \tfrac{1}{\delta_{\text{quat}}}\|\log(q \circ\ q^{\star-1})\|)}_{\text{body orientation}} \tag{13}$$

The first term measures the current displacement between the model and reference CoM positions, $\mathbf{r}$ and $\mathbf{r}^\star$ respectively. The second term is the body orientation mismatch, calculated as the norm of the quaternion "minus operator" between the model and reference quaternions, q and $q^\star$, respectively [10]. δ_{com} and δ_{quat} are the reward tightness hyperparameters. For instance, the reward is zero when the CoM displacement in the first term $\|\mathbf{r} - \mathbf{r}^\star\| > \delta_{\text{com}}$, while it is one when $\|\mathbf{r} - \mathbf{r}^\star\| = 0$, and similarly for the second term. See Supplementary Table 15 for the hyperparameter values used.

In the flight imitation task, we placed the fly legs in a retracted flight position and disabled the leg DoFs and actuators. The retracted leg configuration is stored in the `springref` leg parameters, which were fitted by matching the model's legs to images of flying *Drosophila*. We also removed the antennae and proboscis actuators and excluded their joints from the observations. This reduced the number of observable joint angles and joint velocities to 25. It also reduced the total action dimension to 12. We didn't use vision in this task. The observables (policy inputs) are listed in Supplementary Table 6 and the actions (policy outputs) are shown in Supplementary Table 7. In addition to the standard set of egocentric vestibular and proprioception observables, the policy receives task-specific inputs: the Cartesian CoM displacement and the orientation (quaternion) displacement of the reference trajectory with respect to the model at the current timestep plus 5 timesteps into the future. The displacements are calculated with respect to the current fly model position and orientation and are expressed in the egocentric reference frame of the model. The policy network architecture is shown in Supplementary Table 17. The trained flight imitation policy can be used as a low-level flight controller. In this scenario, the two task-specific inputs (CoM and quaternion displacements) serve as high-level steering control commands.

At training time, we engage action penalization, a DMPO agent feature. It encourages the agent to learn to utilize actions of small magnitude thus keeping the wing motion close to the baseline wing pattern produced by the WPG. The task and agent hyperparameters, including DMPO action penalization, are summarized in Supplementary Tables 14, 15.

[3]Supplementary data: https://doi.org/10.25378/janelia.25309105

1.4 Flight controller reuse, vision-guided flight task configuration

We reused the flight controller trained in the flight imitation task (Figure 2 in main text) in the context of two vision-guided reinforcement learning tasks set as follows. The fly model is required to fly generally in the positive x-direction, to maintain a given target speed and height above terrain, and to avoid collisions with the terrain. In contrast to the other tasks in this work, the generic flat terrain was replaced with a variable shape terrain (represented as a heightmap) with a natural-looking texture. As the policy has no direct access to the flight height and terrain shape, avoiding collisions and assessing the current height requires utilization of the visual input from the two eye cameras. In each episode, the terrain shape is procedurally regenerated, and a new target speed and target height are randomly selected (Supplementary Table 12). The episodes end normally when the time limit is reached, or terminate early when the fly collides with terrain, which results in the loss of all future infinite-horizon rewards.

The task is set in a square 40×40 cm arena. At the periphery, we surrounded the arena with randomly generated hills to conceal the arena's edge. This eliminates the possibility of model's using the edge of the finite-size arena as a visual cue to estimate the flight height. Also, we removed overhead light sources to eliminate the fly's shadows as the shadow size could be exploited to infer the flight height. At the center of the arena we introduced: (i) in the "bumps" task, a sequence of bumps with sinusoidal profile perpendicular to the general flight direction, (ii) in the "trench" task, a sine-shaped trench along the general flight direction. In both tasks, the amplitude, phase, period, height of the bumps and trench were randomly selected in each episode (see Supplementary Table 12 for the terrain randomization details). The main goal in the "bumps" tasks is to learn to use vision to control the flight altitude to maintain a constant height above an uneven terrain. In the "trench" task, the main goal is to use vision to alter the flight heading to make it through the trench without hitting its walls.

In the "bumps" tasks, the (multiplicative) reward consists of several factors as follows:

$$R_{\text{bumps}} = R_{\text{height}} \times R_{\text{speed}} \times R_{\text{dir}} \times R_{\text{heading}} \times R_{\text{gravity}} \tag{14}$$

where the individual reward terms are:

$$\begin{aligned} R_{\text{height}} &= \max(0, 1 - \tfrac{1}{\delta_h}|h - h^\star|) \\ R_{\text{speed}} &= \max(0, 1 - \tfrac{1}{s^\star}\big||\mathbf{v}| - s^\star\big|) \\ R_{\text{dir}} &= \max(0, 1 - \tfrac{1}{s^\star}|v_x - s^\star|) \\ R_{\text{heading}} &= \max(0, 1 - \tfrac{1}{\delta_y} v_y^\dagger) \\ R_{\text{gravity}} &= \max(0, 1 - \tfrac{1}{\pi}\theta_{\text{g}}) \end{aligned} \tag{15}$$

Here, R_{height} is the reward factor measuring how close the current flight height h is to the target height $h^\star$. As in the other tasks, δ_h is the reward tightness hyperparameter (Supplementary Table 12). Similarly, R_{speed} compares the current flight speed $|\mathbf{v}|$ to the target speed $s^\star$ ($\mathbf{v}$ is the fly velocity vector). R_{dir} prescribes a preferred general flight direction by rewarding fly's propagation in the positive x-direction. v_x is the x-component of fly velocity computed in the arena reference frame. R_{heading} requires the fly to keep the body heading parallel to the current velocity direction (e.g., avoid flying sideways). This is achieved by minimizing the lateral velocity component $v_y^\dagger$ computed in fly's egocentric reference frame. R_{gravity} sets a preferred body orientation with respect to the gravity direction (the vertical z-axis) by minimizing θ_{g}, the angle between the current gravity direction vector $\mathbf{g}$ as measured by fly's gravity sensor and the preferred egocentric gravity direction $\mathbf{g}^\star$. This angle is computed as $\theta_{\text{g}} = \cos^{-1}(\mathbf{g} \cdot \mathbf{g}^\star)$. The preferred gravity direction is expressed

in fly's egocentric reference frame as $\mathbf{g}^\star = (\sin(\alpha_{\text{pitch}}), 0, \cos(\alpha_{\text{pitch}}))$, where $\alpha_{\text{pitch}} = 47.5°$ is the default body pitch angle during stable flight in *Drosophila* [2] (Supplementary Table 21).

In the "trench" task, the reward is as in the "bumps" task with an additional term:

$$R_{\text{trench}} = R_{\text{bumps}} \times R_{\text{mid}} \tag{16}$$

The additional term R_{mid} requires the fly to stay close to the midline of the trench and it is written as:

$$R_{\text{mid}} = \begin{cases} \max(0, 1 - \frac{1}{\delta_m}|y - y^\star|) & \text{fly inside trench} \\ 1 & \text{fly before or after trench} \end{cases} \tag{17}$$

This term compares the current fly's y-coordinate (lateral, perpendicular to the general trench direction x) and the current midpoint of the trench, $y^\star$. In the "trench" task, the target height is always smaller than the trench wall height. Also, the trench width and turn amplitude are sampled such that the "trivial" solution of making it through the trench by simply flying straight wouldn't be possible.

In both vision-guided flight tasks, we reused the policy network trained in the flight imitation task (Figure 2 in main text) as a low-level flight controller. We froze the weights of this pre-trained low-level controller network and trained a high-level controller to navigate the flight. In this setup, the low-level controller abstracts away and controls all the fine details of the wing motion (Supplementary Table 11), while the high-level controller only issues low-dimensional steering commands to the low-level controller.

As in the flight imitation task, the fly legs were retracted and their DoFs and actuators disabled. The observations, on the other hand, were altered in two ways. First, we added visual input from the eye cameras as two 32×32 RGB frames. Second, we provided a task-input consisting of the target speed and height, which were randomly sampled in the beginning of each episode. The total observation dimension was 6208, see Supplementary Table 10 for more detail. The RGB visual input was first converted to grayscale, then processed by a convolutional visual module, and then fed into the high-level controller's policy network as a vector of dimension 8, along with the rest of the observables. The steering command output by the high-level controller was concatenated with the proprioception and vestibular (but not visual and task-input) components of the original observation and passed on as input to the low-level controller network. We used a generic convolutional network mostly similar to a single block of ResNet[11]. The architecture of the high-level controller network is shown in Supplementary Table 18. We used the same DMPO agent configuration, including action penalization, as in the flight imitation task (Supplementary Table 14). We trained the vision flight controller, including the convolutional visual module, end-to-end with reinforcement learning. The vision task hyperparameters are summarized in Supplementary Table 12.

1.5 Walking imitation task configuration

The reinforcement learning task is set as follows. In each episode, a reference trajectory is randomly selected from the walking dataset. The model is required to track the CoM position and orientation of the reference fly body, as well as the detailed motion of the legs. The initial model posture and the CoM position and velocity are set equal to the reference in the first step of the selected trajectory. The episode ends when the end of the trajectory is successfully reached or terminates early if the model CoM is displaced from the reference by more than 0.3 cm (one body length.)

We used a multiplicative version of the imitation reward mostly similar to [12, 13]. At every timestep, the reward measures the similarity between the current model and reference pose, position, and orientation. The reward function is formulated as a product of (not normalized) Gaussian functions, one Gaussian for each quantity compared, e.g. a joint angle, the CoM position, etc. As the Gaussians are not normalized, each of them is in the range $[0, 1]$, and so is the total multiplicative reward. There is no unique way to construct this imitation reward function and some of the terms could be redundant/overlapping. At each time step, the reward R is calculated as:

$$R = \exp\big(- E_{\text{com}} - E_{\text{quat}} - E_{\text{qvel}} - E_{\text{ee}}\big) \tag{18}$$

where the four terms expressing different aspects of the pose tracking objective are:

$$\begin{aligned}
E_{\text{com}} &= \frac{1}{2\sigma^2_{\text{com}}} ||\mathbf{r}_{\text{com}} - \mathbf{r}^{\star}_{\text{com}}||^2 \quad \text{center-of-mass position} \\
E_{\text{quat}} &= \frac{1}{2\sigma^2_{\text{quat}}} \sum_{i=1}^{N_{\text{joints}}} ||\log(q_i \circ q_i^{\star -1})||^2 \quad \text{joint quaternions} \\
E_{\text{qvel}} &= \frac{1}{2\sigma^2_{\text{qvel}}} \sum_{i=1}^{N_{\text{joints}}} (v_i - v_i^{\star})^2 \quad \text{joint velocities} \\
E_{\text{ee}} &= \frac{1}{2\sigma^2_{\text{ee}}} \sum_{i=1}^{6} ||\mathbf{r}_i - \mathbf{r}_i^{\star}||^2 \quad \text{end-effector positions}
\end{aligned} \tag{19}$$

E_{com} measures the similarity between the current model and reference Cartesian CoM positions, $\mathbf{r}_{\text{com}}$ and $\mathbf{r}^{\star}_{\text{com}}$. E_{quat} measures the similarity between the model and reference joint quaternions q_i and $q_i^{\star}$ for all joints, including the root joint. Joint quaternions encode both the joint angle and the direction of the joint rotation axis for hinge joints (or body orientation for the root joint.) E_{qvel} expresses the similarity between the model and reference joint velocities, v_i and $v_i^{\star}$. E_{ee} compares the Cartesian positions of the end-effectors (six leg tips), $\mathbf{r}_i$ and $\mathbf{r}_i^{\star}$. Each one of the four error E terms has its own reward tightness (scale) hyperparameter, expressed as a standard deviation σ. See Supplementary Table 16 for the hyperparameter values used.

In the walking imitation task, we set the wings in the retracted position. We removed the actuators of the wings, proboscis, antennae. We retained their DoFs in the model but excluded these DoFs from the policy observations. We didn't use vision in this task. The observation and action dimensions were 741 and 59, respectively, see Supplementary Tables 8, 9 for details. The policy network architecture is shown in Supplementary Table 19. We attached MuJoCo adhesion actuators to leg tips to simulate fly's adhesive pads. The control semantics for the adhesion actuators is the required adhesion force, ranging between zero to one fly body weight for each leg. The rest of the actuators were position actuators receiving target joint angles as control from the policy. We didn't use any data related to adhesion during walking and didn't constrain the model as to how to use the adhesion actuators (e.g., there are no adhesion terms in the reward.) We observed, however, that the agent generally preferred to activate the adhesion while the legs were in stance to increase friction with the ground. In this task, we did not use the DMPO action penalization because the full-body imitation reward automatically constrains the policy outputs (target joint angles) to the proper ranges.

As in the flight imitation task, in addition to the standard set of egocentric vestibular and proprioception observables, the policy receives task-specific inputs: the Cartesian CoM displacement and the orientation (quaternion) displacement of the reference trajectory with respect to the model

at the current timestep plus 64 timesteps into the future. In contrast to the flight tasks, the actions output by the policy are not fed to the actuators directly but first undergo filtering (averaging) in time with time constants `joint_filter` and `adhesion_filter` for joint and adhesion actuators, respectively (Supplementary Table 16). As a result, the current actuator target joint angles (or target force in adhesion actuators) are generally different from the current policy control output and therefore considered as the actuator's internal state. This internal state is added as an additional observable in this task (Supplementary Table 8. As in the flight imitation task, the trained walking policy can be used as a low-level walking controller with the two task-specific inputs (CoM and quaternion displacements) serving as high-level steering control commands.

1.6 Simulation speed

The timing of different components within one control timestep of the simulation is shown in Supplementary Table 5. We simulated the different fly model behaviors on a single core of Intel Xeon CPU E5-2697 v3 @ 2.60GHz. The total simulation control step time is broken down into its components as *Total step time* = *Policy* + *RL env* + *MuJoCo control*, where *Policy* is the forward pass through the policy network, *RL env* is the dm_control python RL environment, and *MuJoCo control* is the total of MuJoCo physics timesteps per one RL environment control timestep [1]. The policy forward pass was run on CPU, same as in actors during training. % real time is shown for both the total simulation timestep and for the MuJoCo component alone. The averages are calculated from 10,000 simulation steps with std-to-mean ratios being on the order of $\sim$0.01. For vision-guided flight, "bumps" task times are shown; "bumps" and "trench" tasks perform similarly; visual input was rendered on an NVIDIA Titan Xp GPU.

In addition, note that the simulation speed can be improved further:

- When not flying, a much larger time-step can be used.
- When flying, contacts can (sometimes) be ignored.
- MuJoCo simulations can be easily duplicated across threads.
- As of MuJoCo 3.0, simulation is supported on GPU and TPU accelerators which can achieve much higher throughput, especially for RL training, as both the physics and network are on the same processor. We have not yet attempted to run our environments on accelerators as this would require rewriting the environment logic (standard Python is bound to CPU).

2 Supplementary Tables

Supplementary Table 1: Empirical masses of the fly body parts. Averaged over 52 female flies.

Body part	Mass (mg)
head	0.15
thorax	0.34
abdomen	0.38
leg (each)	0.0162
wing (each)	0.008
fly total	0.983

Supplementary Table 2: Actuators of the fly model in its default configuration. Task-specific model modifications (e.g., disabling legs for flight) may include removing some of the actuators. By default, each actuator actuates one DoF. In the "position coupled" category, one actuator actuates several DoFs coupled by a MuJoCo tendon.

**Actuator type**	**Located at**	**# of actuators**
torque	wings	6
position	neck	3
	proboscis	5
	antennae	6
	legs (excluding tarsi)	42
position, coupled by tendon	tarsi	6
(several DoFs per actuator)	abdomen	2
adhesion	leg tips	6
	labrum	2
total		78

Supplementary Table 3: Sensory system of the fly model in its default configuration. Task-specific model modifications (e.g., disabling legs for flight) may include disabling some of the sensors and altering the dimensions of the proprioception observables. All observables are calculated in fly's egocentric reference frame. The actuator activation state units are the same as the corresponding actuator control units.

Category	Sensor	Units	Array shape
vision	right eye camera	unitless RGB	(32, 32, 3)
	left eye camera		(32, 32, 3)
vestibular	velocimeter	$\mathsf{cm/s}$	3
	accelerometer	$\mathsf{cm/s^2}$	3
	gyro (rotational velocity)	$\mathsf{rad/s}$	3
	gravity direction	cm	3
proprioception	joint angles	rad	102
	joint velocities	$\mathsf{rad/s}$	102
	actuator activation state	same as control	78
	end-effector positions	cm	(7, 3)
mechanoreception	leg force sensors	dyn	(6, 3)
	leg touch sensors	dyn	6

Supplementary Table 4: Correspondence between the sensory system components in *Drosophila* and in our fly model.

Drosophila sense organ, receptor	Sensory modality	Purpose, detection, sensing	Corresponding fly model sensor	References
Compound eyes	Light, photoreception	Self-movement (optic flow, optomotor response), looming stimuli	Eye cameras, velocimeter, gravity direction	[14, 15]
Ocelli	Light, photoreception	Dorsal light response, body orientation, postural correction	*As above*	[14, 15, 16]
Halteres (modified 2nd wing pair)	Mechanoreception	Coriolis forces during flight; balance/equilibrium organ	Gyro, accelerometer	[15, 17]
Leg campaniform sensilla	Mechanoreception	Load/gravity	Force, touch, gravity direction	[18]
Johnston's organ (2nd antennal segment)	Mechanoreception (exteroception)	Gravity, air flow, sound (courtship song)	Gravity direction, velocimiter, sound not implemented	[19, 16, 20]
Neck proprioceptors (prosternal organ, CO)	Mechanoreception (proprioception)	head position; postural correction	Joint angles, joint velocities	[15]
Wing base tegula	Mechanoreception via a hair plate and campaniform sensilla	Wing position (proprioception)	*As above*	[21]
Femoral chordotonal organ (FeCO)	Mechanoreception	Flexion, extension of the tibia (proprioception); vibration (exteroception)	*As above*	[22]
Coxa and trochanter hair plates	Mechanoreception	Registering extreme positions of the legs (proprioception)	*As above*	[23, 24]
Wing base campaniform sensilla	Mechanoreception	Wing twist, wing load	*Not implemented*	[25, 26]
Wing veins campaniform sensilla	Mechanoreception	Aeroelastic deformations of wing	*Not implemented*	[25, 26]
Wing margin chemosensory bristles	Chemoreception	Airborne molecules (odor plumes, pheromones)	*Not implemented*	[27]
Arista (4th-6th antennal segments)	Hygro-, chemo-, thermoreception	Odor, pheromone plumes, temperature gradients	*Not implemented*	[28]
Labrum and tarsal gustatory receptors	Hygro- and chemoreception	Chemical compounds in the substrate	*Not implemented*	[29]

Supplementary Table 5: Breakdown of average times of one control timestep for different fly model behaviors simulated on a single core of Intel Xeon CPU E5-2697 v3 @ 2.60GHz. All times are in units of ms.

Component:	Policy	RL env	MuJoCo		Total	Real time	% real time	
Behavior:			control	physics	step time	simulated	total	MuJoCo
Flight	3.64	3.67	1.11	0.278	8.43	0.2	2.37%	18.0%
Walking	4.31	4.49	4.64	0.464	13.73	2.0	14.6%	43.1%
Vision-guided flight	4.51	9.83	1.03	0.257	15.36	0.2	1.30%	19.5%

Supplementary Table 6: Observations in the flight imitation task. All observables are calculated in fly's egocentric reference frame.

**Category**	**Observables (policy inputs)**	**Units**	**Array shape**
vestibular	velocimeter	cm/s	3
	accelerometer	cm/s^2	3
	gyro	rad/s	3
	gravity direction	cm	3
proprioception	joint angles	rad	25
	joint velocities	rad/s	25
task inputs	reference CoM displacement	cm	(6, 3)
(steering commands)	reference orientation displacement	unitless quaternion	(6, 4)
total obs. dimension			104

Supplementary Table 7: Actions in the flight imitation task.

**Actions (policy outputs)**	**Array shape**
head	3
wings	6
abdomen	2
WPG frequency control	1
total action dimension	12

Supplementary Table 8: Observations in the walking imitation task. All observables are calculated in fly's egocentric reference frame. The actuator activation state units are the same as the corresponding actuator control units.

Category	Observables (policy inputs)	Units	Array shape
vestibular	velocimeter	cm/s	3
	accelerometer	cm/s^2	3
	gyro (rotational velocity)	rad/s	3
	gravity direction	cm	3
proprioception	joint angles	rad	85
	joint velocities	rad/s	85
	actuator activation state	same as control	59
	end-effector positions	cm	(7, 3)
mechanoreception	leg force sensors	dyn	(6, 3)
	leg touch sensors	dyn	6
task input	reference CoM displacement	cm	(65, 3)
(steering commands)	reference orientation displacement	unitless quaternion	(65, 4)
total obs. dimension			741

Supplementary Table 9: Actions in the walking imitation task.

Actions (policy outputs)	Array shape
head	3
abdomen	2
legs	(6, 8)
leg adhesion	6
total action dimension	59

Supplementary Table 10: Observations in the vision-guided flight task. All observables are calculated in fly's egocentric reference frame.

Category	Observable (policy input)	Units	Array shape
vision	right eye	RGB (byte)	(32, 32, 3)
	left eye		(32, 32, 3)
vestibular	velocimeter	cm/s	3
	accelerometer	cm/s^2	3
	gyro (rotational velocity)	rad/s	3
	gravity direction	cm	3
proprioception	joint angles	rad	25
	joint velocities	rad/s	25
task input	target flight speed	cm/s	1
	target flight height	cm	1
total observation dimension			6208

Supplementary Table 11: Actions in the vision-guided flight task. The pre-trained low-level controller outputs the full 12-dimensional actions, same as in the flight imitation task.

Action (policy output)	Array shape
head	3
wings	6
abdomen	2
WPG frequency control	1
total action dimension	12

Supplementary Table 12: Vision-guided flight task parameters and ranges of sine bump and trench terrain randomization.

Parameter	Value	Units
`eye_camera_fovy`	150	deg
`eye_camera_size`	32	pixels
`vis_output_dim`	8	
`time_limit`	0.4	s
reward parameters:		
δ_h	0.15	cm
$s^\star$	$1.1\times$`target_speed`	cm/s
δ_y	10	cm/s
δ_m	0.15	cm
flight speed range	[20, 40]	cm/s
flight height range	[0.5, 0.8]	cm
bump terrain parameters:		
period range	[10, 15]	cm
height range	[0.5, 1]	cm
phase range	[0, 2π]	rad
trench terrain parameters:		
period range	[5, 8]	cm
amplitude range	[0.35, 0.6]	cm
phase range	[0, 2π]	rad
width range	$2\times$ amplitude + [0.3, 0.6]	cm
height	1.3	cm
length range	[4, 10]	cm
initial fly distance from trench	[0, 2]	cm

Supplementary Table 13: Distributed training hyperparameters. See implementation for more detail.

Hyperparameter	Value (walking)	Value (flight)
num_actors	32	32
batch_size	256	256
prefetch_size	4	4
min_replay_size	10^4	10^4
max_replay_size	4×10^6	4×10^6
samples_per_insert	15	15
n_step	10	5
num_samples	20	20
policy_optimizer_lr	10^{-4}	10^{-4}
critic_optimizer_lr	10^{-4}	10^{-4}
dual_optimizer_lr	10^{-3}	10^{-3}
target_critic_update_period	200	107
target_policy_update_period	200	101

Supplementary Table 14: DMPO agent hyperparameters. See implementation for more detail.

Hyperparameter	Value (walking)	Value (flight)
epsilon	0.05	0.1
epsilon_mean	5×10^{-5}	2.5×10^{-3}
epsilon_stddev	10^{-7}	10^{-7}
action_penalization	False	True
epsilon_penalty	N/A	0.1
init_log_temperature	10	10
init_log_alpha_mean	10	10
init_log_alpha_stddev	10^3	10^3
per_dim_constraining	True	True

Supplementary Table 15: Flight imitation task hyperparameters. See implementation for more details.

**Parameter**	**Value**	**Units**
physics_timestep	5×10^{-5}	s
control_timestep	2×10^{-4}	s
joint_filter	0	s
adhesion_filter	N/A	s
future_steps	5	
time_limit	0.6	s
terminal_com_dist	2.0	cm
reward parameters:		
δ_{com}	0.4	cm
δ_{quat}	3.14	rad

Supplementary Table 16: Walking imitation task hyperparameters.

Parameter	Value	Units
`physics_timestep`	2×10^{-4}	s
`control_timestep`	2×10^{-3}	s
`joint_filter`	0.01	s
`adhesion_filter`	0.007	s
`claw_friction`	1.0	
`future_steps`	64	
`time_limit`	10.0	s
`terminal_com_dist`	0.3	cm
reward parameters:		
σ_{com}	0.0785	cm
σ_{qvel}	53.780	rad/s
σ_{ee}	0.0735	cm
σ_{quat}	1.225	rad

Supplementary Table 17: Flight policy network architecture. Layer sizes indicated in parentheses.

input: observations	
Linear (256)<br>LayerNorm<br>tanh<br>Linear (256)<br>ELU<br>Linear (256)<br>ELU	
Linear (12)	Linear (12)<br>softplus
output: action mean	*output*: action std

Supplementary Table 18: Architecture of the high-level controller network for the vision-guided flight task. Visual input is pre-processed by a convolutional visual module and concatenated with the rest of the observations. Layer sizes indicated in parentheses. This high-level controller outputs a steering command of dimension $7 \times (\text{future\_steps} + 1)$. The steering command is then concatenated with the proprioception and vestibular (but not task-input and visual) components of the observations and sent as input to the low-level controller network, which is the policy from the flight imitation task (Supplementary Table 17).

input: observations	
Linear (256)<br>LayerNorm<br>tanh<br>Linear (256)<br>ELU<br>Linear (128)<br>ELU	
Linear (42)	Linear (42)<br>softplus
output: action mean	*output*: action std

Supplementary Table 19: Walking policy network architecture. Layer sizes indicated in parentheses.

input: observations	
Linear (512)<br>LayerNorm<br>tanh<br>Linear (512)<br>ELU<br>Linear (512)<br>ELU<br>Linear (512)<br>ELU	
Linear (59)	Linear (59)<br>softplus
output: action mean	*output*: action std

Supplementary Table 20: Fluid force model coefficients. See Section 1.2 for definition. The coefficients are stored in the `fluidcoef` MuJoCo attribute, in this order.

Index	Coefficient	Description	Value
0	C_D^{blunt}	Blunt drag	1.0
1	C_D^{slender}	Slender drag	0.5
2	C_D^{angular}	Angular drag	1.5
3	C_K	Kutta lift	1.7
4	C_M	Magnus lift	1.0

Supplementary Table 21: Flight physics parameters.

Parameter	Value	Units
`fluidcoef`	[1.0, 0.5, 1.5, 1.7, 1.0]	unitless
`gainprm`	[18, 18, 18]	dyn cm
`damping`	0.007769	dyn s
`body_pitch_angle`	47.5	deg
`stroke_plane_angle`	0	deg
`base_freq`	218	Hz

Supplementary References

[1] S. Tunyasuvunakool, A. Muldal, Y. Doron, S. Liu, S. Bohez, J. Merel, T. Erez, T. Lillicrap, N. Heess, and Y. Tassa, "dm_control: software and tasks for continuous control," *Software Impacts*, vol. 6, p. 100022, 2020.

[2] F. T. Muijres, M. J. Elzinga, J. M. Melis, and M. H. Dickinson, "Flies evade looming targets by executing rapid visually directed banked turns," *Science*, vol. 344, no. 6180, pp. 172–177, 2014.

[3] A. Andersen, U. Pesavento, and Z. J. Wang, "Analysis of transitions between fluttering, tumbling and steady descent of falling cards," *Journal of Fluid Mechanics*, vol. 541, pp. 91–104, 2005.

[4] G. J. Berman and Z. J. Wang, "Energy-minimizing kinematics in hovering insect flight," *J. Fluid Mech.*, vol. 582, pp. 153–168, 2007.

[5] Z. Duan, B. He, and Y. Duan, "Sphere drag and heat transfer," *Scientific reports*, vol. 5, no. 1, pp. 1–7, 2015.

[6] G. Stokes, "On the effect of internal friction of fluids on the motion of pendulums," *Trans. Camb. phi1. S0c*, vol. 9, no. 8, p. 106, 1850.

[7] H. Lamb, *Hydrodynamics. Sixth edition.* Cambridge University Press, 1932.

[8] L. Tuckerman, *Inertia factors of ellipsoids for use in airship design*. US Government Printing Office, 1925.

[9] F. T. Muijres, M. J. Elzinga, N. A. Iwasaki, and M. H. Dickinson, "Body saccades of Drosophila consist of stereotyped banked turns," *Journal of Experimental Biology*, vol. 218, pp. 864–875, 03 2015.

[10] J. Sola, "Quaternion kinematics for the error-state kalman filter," *arXiv preprint arXiv:1711.02508*, 2017.

[11] K. He, X. Zhang, S. Ren, and J. Sun, "Deep residual learning for image recognition," 2015.

[12] X. B. Peng, P. Abbeel, S. Levine, and M. Van de Panne, "Deepmimic: Example-guided deep reinforcement learning of physics-based character skills," *ACM Transactions On Graphics (TOG)*, vol. 37, no. 4, pp. 1–14, 2018.

[13] J. Merel, S. Tunyasuvunakool, A. Ahuja, Y. Tassa, L. Hasenclever, V. Pham, T. Erez, G. Wayne, and N. Heess, "Catch & carry: reusable neural controllers for vision-guided whole-body tasks," *ACM Transactions on Graphics (TOG)*, vol. 39, no. 4, pp. 39–1, 2020.

[14] R. Hengstenberg, "Multisensory control in insect oculomotor systems, in visual motion and its role in the stabilization of gaze," *Reviews of Oculomotor Research*, vol. 5, no. 2, p. 285–298, 1993.

[15] N. J. Strausfeld and H. S. Seyan, "Convergence of visual, haltere, and prosternai inputs at neck motor neurons of calliphora erythrocephala," *Cell and Tissue Research*, vol. 240, no. 3, pp. 601–615, 1985.

[16] K. Mimura, H. Tateda, H. Morita, and M. Kuwabara, “Convergence of antennal and ocellar inputs in the insect brain,” *Zeitschrift für vergleichende Physiologie*, vol. 68, no. 3, pp. 301–310, 1970.

[17] A. Fayyazuddin and M. H. Dickinson, “Haltere afferents provide direct, electrotonic input to a steering motor neuron in the blowfly, *Calliphora*,” *The Journal of Neuroscience*, vol. 16, p. 5225, 08 1996.

[18] G. F. Dinges, A. S. Chockley, T. Bockemühl, K. Ito, A. Blanke, and A. Büschges, “Location and arrangement of campaniform sensilla in drosophila melanogaster,” *Journal of Comparative Neurology*, vol. 529, no. 4, pp. 905–925, 2021.

[19] A. Kamikouchi, H. K. Inagaki, T. Effertz, O. Hendrich, A. Fiala, M. C. Göpfert, and K. Ito, “The neural basis of drosophila gravity-sensing and hearing,” *Nature*, vol. 458, no. 7235, pp. 165–171, 2009.

[20] A. Mamiya and M. H. Dickinson, “Antennal mechanosensory neurons mediate wing motor reflexes in flying drosophila,” *The Journal of Neuroscience*, vol. 35, p. 7977, 05 2015.

[21] W. Fudalewicz-Niemczyk, “L’innervation et les organes sensoriels des ailes des dipteres et comparaison avec l’innervation des ailes d’insectes d’autres ordres,” *Acta Zoologica Cracoviensia*, vol. 8, pp. 351–462, 1963.

[22] A. Mamiya, A. Sustar, I. Siwanowicz, Y. Qi, T.-C. Lu, P. Gurung, C. Chen, J. S. Phelps, A. T. Kuan, A. Pacureanu, W.-C. A. Lee, H. Li, N. Mhatre, and J. C. Tuthill, “Biomechanical origins of proprioceptor feature selectivity and topographic maps in the drosophila leg,” *Neuron*, 2023.

[23] R. K. Murphey, D. Possidente, G. Pollack, and D. J. Merritt, “Modality-specific axonal projections in the cns of the flies phormia and drosophila,” *Journal of Comparative Neurology*, vol. 290, no. 2, pp. 185–200, 1989.

[24] D. J. Merritt and R. K. Murphey, “Projections of leg proprioceptors within the cns of the fly phormia in relation to the generalized insect ganglion,” *Journal of Comparative Neurology*, vol. 322, no. 1, pp. 16–34, 1992.

[25] E. S. Cole and J. Palka, “The pattern of campaniform sensilla on the wing and haltere of Drosophila melanogaster and several of its homeotic mutants,” *Development*, vol. 71, pp. 41–61, 10 1982.

[26] M. Dickinson and J. Palka, “Physiological properties, time of development, and central projection are correlated in the wing mechanoreceptors of drosophila,” *Journal of Neuroscience*, vol. 7, no. 12, pp. 4201–4208, 1987.

[27] B. Houot, V. Gigot, A. Robichon, and J.-F. Ferveur, “Free flight odor tracking in drosophila: Effect of wing chemosensors, sex and pheromonal gene regulation,” *Scientific Reports*, vol. 7, no. 1, p. 40221, 2017.

[28] R. F. Foelix, R. F. Stocker, and R. A. Steinbrecht, “Fine structure of a sensory organ in the arista of drosophila melanogaster and some other dipterans,” *Cell and Tissue Research*, vol. 258, no. 2, pp. 277–287, 1989.

[29] C. Montell, “A taste of the drosophila gustatory receptors,” *Current Opinion in Neurobiology*, vol. 19, no. 4, pp. 345–353, 2009. Sensory systems.
